# Evaluating the Sporicidal Activity of Disinfectants against *Clostridium difficile* and *Bacillus amyloliquefaciens* Spores by Using the Improved Methods Based on ASTM E2197-11

**DOI:** 10.3389/fpubh.2018.00018

**Published:** 2018-02-05

**Authors:** Marie Christine Uwamahoro, Richard Massicotte, Yves Hurtubise, François Gagné-Bourque, Akier Assanta Mafu, L’Hocine Yahia

**Affiliations:** ^1^Laboratory of Innovation and Analysis of Bioperformance, Ecole Polytechnique de Montreal, Montreal, QC, Canada; ^2^Centre Intégré de Santé et de Services Sociaux de Lanaudière, Joliette, QC, Canada; ^3^Biotechnologies Ulysse, Trois-Rivières, QC, Canada; ^4^Saint-Hyacinthe Research and Development Centre, Agriculture and Agri-Food Canada, St-Hyacinthe, QC, Canada

**Keywords:** *Clostridium difficile*, *Bacillus amyloliquefaciens*, disinfectant, spores, sporicidal

## Abstract

Spore-forming pathogenic bacteria, such as *Clostridium difficile*, are associated with nosocomial infection, leading to the increased use of sporicidal disinfectants, which impacts socioeconomic costs. However, *C. difficile* can be prevented using microorganisms such as *Bacillus amyloliquefaciens*, a prophylactic agent that has been proven to be effective against it in recent tests or it can be controlled by sporicidal disinfectants. These disinfectants against spores should be evaluated according to a known and recommended standard. Unfortunately, some newly manufactured disinfectants like Bioxy products have not yet been tested. ASTM E2197-11 is a standard test that uses stainless steel disks (1 cm in diameter) as carriers, and the performance of the test formulation is calculated by comparing the number of viable test organisms to that on the control carriers. Surface tests are preferable for evaluating disinfectants with sporicidal effects on hard surfaces. This study applies improved methods, based on the ASTM E2197-11 standard, for evaluating and comparing the sporicidal efficacies of several disinfectants against spores of *C. difficile* and *B. amyloliquefaciens*, which are used as the test organisms. With the improved method, all spores were recovered through vortexing and membrane filtration. The results show that chlorine-based products are effective in 5 min and Bioxy products at 5% w/v are effective in 10 min. Although Bioxy products may take longer to prove their effectiveness, their non-harmful effects to hospital surfaces and people have been well established in the literature.

## Introduction

Hospital-acquired infections are caused by a variety of pathogens, including *Clostridium difficile*, which is identified as the principal causative agent of nosocomial diarrhea (1–3). Patients at high risk are those with a history of antibiotic treatment, prolonged hospitalization, and the elderly (4, 5). However, bacterial spreading further into the community has been reported (6). *C. difficile* produces resistant spores less susceptible to biocidal products than vegetative form (7). These spores can last for long periods on surfaces. Their resistance is mainly acquired through spore structures, such as spore coat, dehydrated spore core, small acid-soluble spore proteins (SASPs), and inner membrane mostly immobile and impermeable (8, 9). A small number of spores of *C. difficile* is sufficient to initiate an infection (10, 11). There have been very low level outbreaks of *C. difficile* diarrhea in Québec, due to the preventive and control measures in place, as well as additional funds deployed to control this pathogenic bacterium. However, outbreaks of *Clostridium difficile*-associated disease (CDAD) occur on an episodic basis in most health care facilities (12).

Commercial sporicidal formulations have been constantly increased due to the emergence of *C. difficile* infection, as significant nosocomial disease. Current disinfectant products, e.g., oxidizing agents (hydrogen peroxide) and chlorine-releasing agents (sodium hypochlorite, sodium dichloroisocyanurate) (13–15), are continually being improved and new ones are being developed (16–20).

Three main categories of sporicidal chemicals are alkylating agents, oxidizing agents, and chlorine-releasing agents (15). Chemicals belonging to the latter two classes have been reported to be effective against bacterial spores within a few minutes. The Canadian hospital environment requires a product with maximum efficiency in 10 min or less, to meet the requirements of bed management (13), but most of those disinfectants have associated drawbacks (19, 21, 22).

Chlorine-based disinfectants such as sodium hypochlorite are recommended for the environmental control of *C. difficile* spores (23). Despite their low cost and ready availability (21), the long-term use of hypochlorite has been found to be environmentally destructive (24), and its effectiveness, as well as its secure handling, is inconclusive (7). Sodium dichloroisocyanurate (NaDCC) is also a chlorine-releasing agent. Although it is affordable and effective against *C. difficile* spores, sodium dichloroisocyanurate (NaDCC) is associated with a disagreeable odor and can be irritating to the respiratory tract (22). Like sodium hypochlorite, NaDCC damages surfaces and material, significantly increasing the cost of disinfection, because the surfaces must be repainted or changed.

A proper comparison of sporicidal data from the literature is impeded by a number of reasons, including the use of a range of standard tests, various spore preparation methods, and bacterial strains. When assessing disinfectant effectiveness, organisms specified by the standards need to be employed.

Non-pathogenic *Bacillus subtilis* spores are usually used as indicators of efficient disinfection, while spores of *C. difficile* are used for evaluating the efficacy of disinfectants with *C. difficile* claim (25). *Bacillus* and *Clostridium* spores have been studied and shown the greatest resistance to antiseptics and disinfectants among other bacterial spores.

Spore resistance mechanisms are best understood in the *Bacillus* species, most specifically *B. subtilis* spores (18, 25). However, considering the relatedness of *B. subtilis* and *B. amyloliquefaciens*, spores of *B. amyloliquefaciens* can be used in assessing the efficacy of disinfectants, as they are closely related, and not easily distinguished from *B. subtilis* (26). Interestingly, Geeraerts et al. found that *B. amyloliquefaciens* is a prophylactic agent against *C. difficile* (27).

The use of ASTM carrier tests leads to better estimates, i.e., they make it possible to evaluate the effectiveness of disinfectants on a surface and, thus, in real-life setting. This standard E-2197-11 of ASTM International uses disks of stainless steel (1 cm in diameter) as carriers, and the performance of the test formulation is calculated by comparing the number of viable test organisms to that on the control carriers (28). The current study aimed at comparing the efficacy of four commonly used chemical disinfectants and two newly manufactured disinfectants against spores of the ATCC 43593 strain of *C. difficile* and BS-01 *B. amyloliquefaciens* U50, using the improved methods based on ASTM E2197-11standard.

## Materials and Methods

### Chosen Disinfectants

Table 1 presents the six disinfectants used in this study, of which four are surface disinfectants routinely used in Québec. Of those, three are chlorine-based (sodium hypochlorite (6,000 ppm), sodium hypochlorite (3.65% w/w), and sodium dichloro-striazinetrione (5,000 ppm), and the fourth is an AHP (accelerated hydrogen peroxide)-based product which is recognized by Health Canada as a sporicide (29). The two remaining products in this study are newly manufactured disinfectants. The Bioxy H is a non-foaming powdered product that, when dissolved in water, generates three active disinfectants (peroxy acetic agent, hydrogen peroxide, and two quaternary chains of fourth generation at a near-neutral pH). The second, Bioxy+, is a powdered product that, when dissolved in water, generates two active disinfectants (peroxy-acetic agent and hydrogen peroxide) at a near-neutral pH, making it safe for human use. The sporicidal efficacies of these two new disinfectants have not yet been tested to Canadian standards.

**Table 1:** Tested disinfectants.

Active substances	Commercial name	Concentration
Sodium hypochlorite	Complete 6000 Liquid	6,000 ppm
Sodium hypochlorite	Complete Gel	3.65% w/w
Sodium dichloro-s-triazinetrione	Zochlor	5,000 ppm
Hydrogen peroxide	Rescue Sporicidal Liquid	4.5%
Peroxy acetic agent, hydrogen peroxyde and quaternary ammonium fourth generation	Bioxy H	5%
	Bioxy H	2%
Peroxy acetic agent and hydrogen peroxide	Bioxy+	2%

### Solution Preparations

Sodium hypochlorite (6,000 ppm), sodium hypochlorite (3.65% w/w), and hydrogen peroxide (4.5%) are provided in ready-to-use liquid form. Sodium dichloro-s-triazinetrione is supplied in tablet form, from which a 5,000 ppm chlorine solution was prepared by mixing five tablets with 1 L of sterile deionized water, followed by mixing for 20 min. Both Bioxy H and Bioxy+ were in powder form, and were mixed with sterile deionized water for 10 min prior to use. Two concentrations of Bioxy H (2 and 5%) and one concentration of Bioxy+ (2%) were used. Phosphate-buffered saline tablets were purchased from Sigma-Aldrich and one tablet is dissolved in 200 mL of deionized water.

### Spores Preparation

For the test to be valid, the titer of the spore suspensions had to achieve a minimum mean of 1 × 10^5^ spores per carrier. Two spore-producing bacteria, BS-01 *B. amyloliquefaciens* U50 and *C. difficile* (ATCC^®^ 43593™), were used in this study. High-titered spore suspensions were generated using standard methods*. B. amyloliquefaciens* spores suspensions were prepared based on the standards ASTM E2197-11, while for *C. difficile* spore suspensions, the preparation was based on modified ASTM 2839-11 and ASTM E2197-11 standards (28, 30–32).

Vegetative *C. difficile* bacterial cells were stored on protected beads, and streaked on pre-reduced CBA (Columbia Blood Agar) to grow colonies. The incubation was carried out in plastic bags, anaerobic atmosphere generation bags were added to generate anaerobic conditions, and nitrogen gas was used to displace the oxygen introduced into the plastic bag. After incubation for 24 h, all colonies on the plates appeared to be uniform for *C. difficile*; a single colony was isolated and cultured into quadrants using sterilize loop on pre-reduced CBA. Vegetative cells of *C. difficile* were grown for 4–10 days under anaerobic conditions at 37°C to induce sporulation. Spore suspensions of high titer (9 log10 spores/mL) and purity (90% spores) were obtained. Phase contrast microscopy was used to evaluate spore formation after 2, 5, and 10 days, following which the spores were harvested. The viable spore stock was 2 × 10^9^. Another single colony was grown aerobically, and no growth was observed (control plate).

*Bacillus amyloliquefaciens* vegetative cells were grown on TSA (tryptic soy agar) at 37°C for 24 h. Then, an isolated colony was cultured on TSA for 96 h to produce spores. Phasecontrast microscopy was used to confirm the rate of sporulation. When the rate of sporulation reached more than 90%, the plate was sterilized at 70°C for 20 min to ensure that the vegetative cells were killed. A count was subsequently made to confirm that sufficient cells were obtained for the tests. The viable spore stock for *B. amyloliquefaciens* was 2.7 × 10^10^.

A spore suspension designed to give a concentration of 1.4 × 10^9^ spores/mL was made for both strains in the present work.

### Neutralization Solution

Chlorine-based formulations were neutralized by a combination of 1% sodium thiosulfate, 0.85% saline solution, and 0.1% Tween 80 (Bioshop, Burlington, ON, Canada) (19). The AHP-based and Bioxy products were neutralized by the same mixture, which also contained 0.1% beef extract.

### Testing of Sporicidal Activity

We adopted methods based mainly on the Standard Quantitative Disk Carrier Test Method. Changes were made to the test method which normally uses spores of *B. subtilis* and *C. sporogenes*. In our research, we used pertinent clinical strain: *C. difficile*, while *B. amyloliquefaciens* was used rather than *B. subtilis*. The assays were performed in the laboratory of Biotechnologies Ulysse, Trois-Rivières, Québec, Canada. Six different chemicals were tested in this study. Sodium hypochlorite (6,000 ppm), sodium hypochlorite (3.65% w/w), sodium dichloro-s-triazinetrione (5,000 ppm), hydrogen peroxide (4.5%), Bioxy H (2 and 5%), and Bioxy+ (2%). Stainless steel carrier disks (1 cm diameter and approximately 0.7 mm thick) were placed in Petri dishes, and each carrier disk was inoculated by applying 10 mL of the test spore suspension at the center of the disk. The dishes were kept in a laminar flow cabinet, at ambient temperature, for 90 min, left open to permit drying. After drying, each inoculated carrier was put into a sterile glass vial with the inoculated side up. Also, 50 µL of disinfectant was then added to the vial covering the whole surface of the carrier disk.

In addition, 10 mL of neutralizing solution was used for neutralization after the recommended manufacturers’ disinfectant contact time. After a 1-min neutralization period, three washes of 10 mL of PBS were performed, and a further 30 mL of PBS was used to rinse the sides of the filter. The surviving organisms were recovered by 30-s vortex and membrane filtration. The eluent was passed through a membrane filter, which was removed and plated onto TSA or Columbia anaerobic sheep blood agar for *B. amyloliquefaciens* and *C. difficile*, respectively.

The assay for *B. amyloliquefacien* was incubated overnight at 37°C under aerobic conditions, while that for *C. difficile* was incubated at 37°C for 24–48 h under anaerobic conditions. The colony-forming units (cfu’s) on the plates were recorded. Log_10_ reductions of the surviving test organisms are calculated and compared with those of the control carriers; ≥5 log_10_ reduction was the performance criterion in the present work.

### Controls

Control carrier disks were not treated in the same way as treated carrier disks, because when applying the same principle as described in the standard ASTM E2197-11, it was not possible to count colonies on the plates. Therefore, a plate counting method was used for counting colonies. Three control disks, with 50 mL of phosphate buffered saline (PBS), were used in each experiment. After neutralization, the content was vortexed; 10^−1^ and 10^−2^ serial dilutions of the resulting eluate were performed and incubated on the culture media under the required conditions for cfu enumeration. Spore concentration per mL and log reduction were then calculated.

### Statistical Analysis

Assays were carried out in pentaplicate and repeated three times. SD analyses were performed.

## Results

### Control

**Figure 1:**
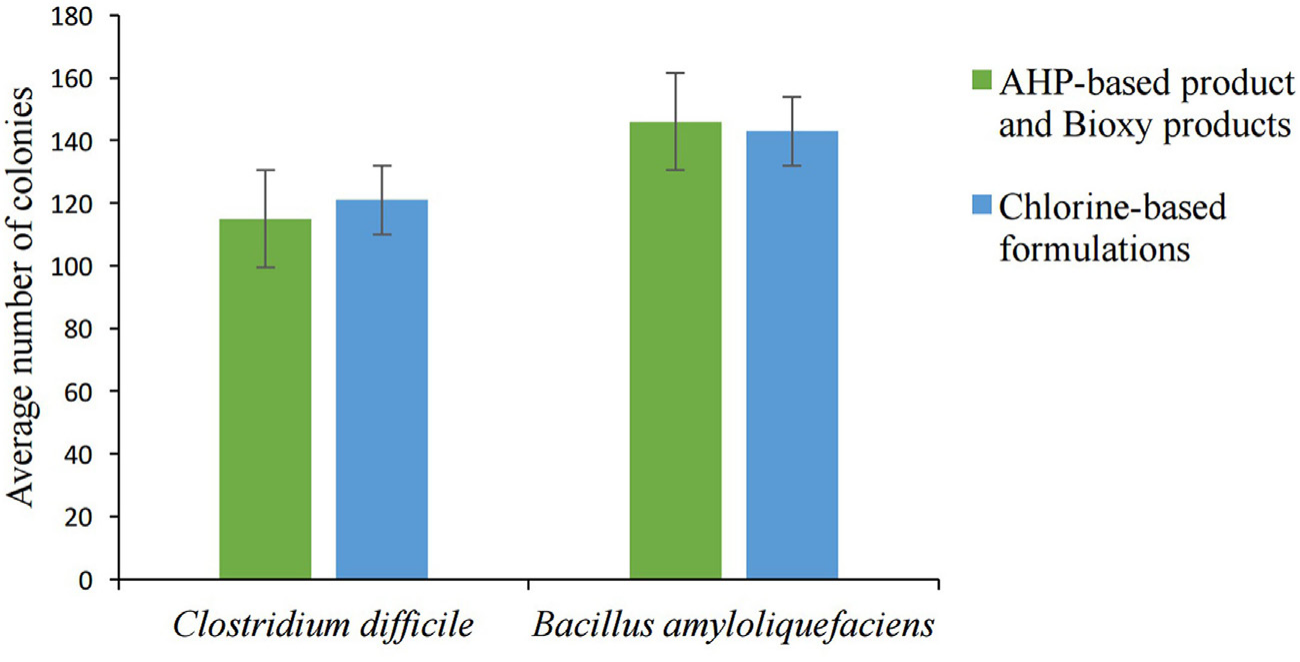
Average number of colonies grown on the plates at 1:100 dilution (error bars represent SE of the mean).

The spore concentration was adjusted to 1.4 10^7^ spores/mL. The average number of colonies for both *C. difficile* and *B. amyloliquefaciens* bacteria at a 10^−2^dilution solution is shown in Figure 1. The colonies in the culture stock solution and at a 10^−1^ dilution solution were greater than 330 and, therefore, impossible to count.

### Sporicidal Tests

**Figure 2:**
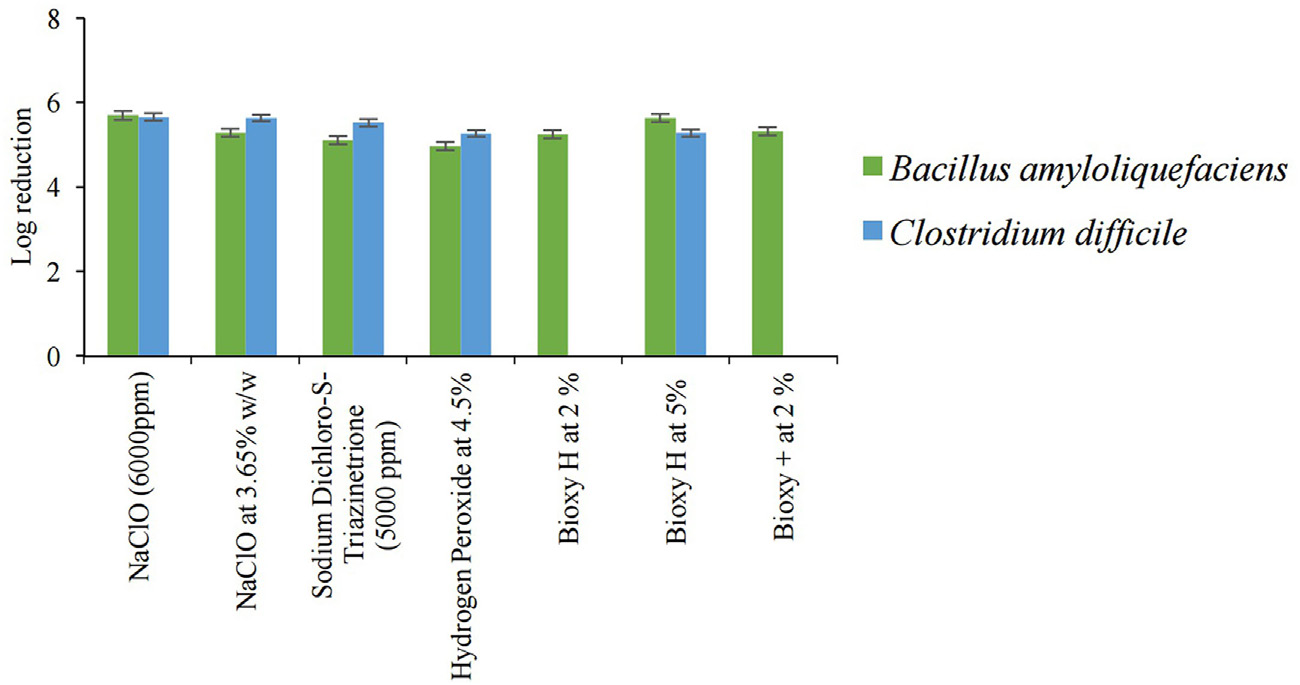
Sporicidal activities of all disinfectants tested against spores of *Bacillus amyloliquefaciens* in 5 min and spores of *Clostridium difficile* in 5 min for chlorine-based products, and in 10 min for AHP-based product and Bioxy products (error bars represent SE of the mean).

The sporicidal activities of all disinfectants were tested for 5 min for *B. amyloliquefaciens* spores. For *C. difficile* spores, chlorine-based products were tested for 5 min, whereas for AHP-based product and Bioxy products are tested for 10 min. Figure 2 shows the results of the sporicidal activity of all tested disinfectants against spores of *C. difficile* (ATCC 43593) and BS-01 *B. amyloliquefaciens* U50. As illustrated in Figure 2, all disinfectants, except 4.5% hydrogen peroxide, were effective in inactivating *B. amyloliquefaciens* spores after 5 min. Log_10_ reductions of disinfectants were 5.693 for 6,000 ppm NaClO, 5.277 for 3.65% w/w NaClO, 5.103 for sodium dichloro-s-triazinetrione, 5.246 for 2% Bioxy H, 5.635 for 5% Bioxy H, 5.316 for 2% Bioxy+, and 4.968 for 4.5% hydrogen peroxide.

All chlorine-based products were effective against spores of *C. difficile* after 5 min. Log_10_ reductions were as follows: 5.6515 for 6,000 ppm NaClO, 5.63 for 3.65% w/w NaClO, and 5.519 for sodium dichloro-s-triazinetrione. Hydrogen peroxide was effective only in inactivating spores of *C. difficile* after 10 min, giving a log_10_ reduction of 5.264. For the Bioxy products, only 5% Bioxy H was effective against *C. difficile* spores after 10 min where the log_10_ reduction was 5.275. Both Bioxy H and Bioxy+ at 2% were found ineffective at inactivating spores of *C. difficile* after 10-min contact time.

## Discussion

Our findings demonstrate that chlorine-based products are efficient at inactivating spores of both *B. amyloliquefaciens* and *C. difficile*. In most cases, *C. difficile* spores were more sensitive. This is in agreement with the results of the recent study which showed that spores of *C. difficile* were more susceptible than *B. subtilis* spores (20). However, previous work has found that *C. difficile* spores were less sensitive to all chlorine-based products tested (19). Despite their sporicidal effect, sodium hypochlorite and hydrogen peroxide are reported to be corrosive on stainless and galvanized steel surfaces (16, 21). This aggressive nature of the older disinfectants may result in higher disinfection costs because surfaces need eventual repainting or replacing. Furthermore, it was reported that sodium hypochlorite reacts with melamine and arborite surfaces, leading to a loss of effectiveness of the disinfectant (14). In our research, we noticed that hydrogen peroxide requires long contact time, becoming effective only after 10 min. This finding has been observed in another study (21). It is important to note that peroxide alone is corrosive because of the acidic pH of the product. Hydrogen peroxide (itself a disinfectant) is released by the powerful oxidizer peracetic acid (PAA), which biodegrades to carbon dioxide, water, and oxygen. Notwithstanding its high sporicidal action, PAA presents the following downside, among others: corrosiveness and strong odor.

There are, at present, new reformulations of sodium hypochlorite and hydrogen peroxide (17), and new disinfectants are being developed for high-level disinfection, particularly against *C. difficile* spores; whence Bioxy products, powdered PAA that produce up to 10% PAA *in situ* when dissolved in water. They are odorless, stable under storage, and safe to transport and handle. They have been proposed in a recent publication as alternatives to liquid PAA and other peroxides, both liquid and powder chlorinated products, quats, aldehydes, iodophors, and alcohols (33). Another work has shown that Bioxy products have great wettability and surface tension, making them powerful disinfectants (17). Recently published data demonstrate the effectiveness of Bioxy products against various microorganisms and spores of *C. difficile* (33).

Moreover, results obtained in this study indicate that 5% Bioxy H has a strong sporicidal activity, i.e., it is effective against *C. difficile* spores in 10 min and has a log reduction of 5.275. These findings are in agreement with results obtained by Dagher et al., who observed that a 5% w/v solution of Bioxy effected a 5.36 log reduction in *C. difficile* spores after 10-min contact time (33). We also noted that 2% Bioxy H and 2% Bioxy+ were not effective against the spores of both bacteria. However, this concentration proved effective against several microorganisms in 10 min (33). This could suggest that at low concentration, Bioxy products are effective against vegetative cells of bacteria whereas at high concentration they are likely more effective against spores.

Only a few available studies have compared sporicidal activities of disinfectants, using the same test conditions such as standard tests, spore preparation methods, and bacterial strains (20). But no study comparing the efficacy of Bioxy products with that of other disinfectants has been performed to date. In the present study, we compared the sporicidal activities of commonly used disinfectants with that of newly manufactured Bioxy products. A study has shown that surface tests are preferable to suspension tests when evaluating the activity of sporicides on surfaces (34). However, in a recent study, authors did not find any significant differences in the sporicidal effectiveness between surface tests (ASTM E2197 and AOAC MB-15-03) and suspension tests (BS EN14347 and BS EN13704), then they noticed that BS EN 14347 tests were better than the other tests (20). This could be because the enumeration of viable count for all tests was performed according to BS EN 14347 only.

In our study, the improved method worked well on all treated carriers, and all spores could be recovered through vortexing and membrane filtration. All the tests were carried out without adding soil load to the spore suspensions. The results presented correspond to those obtained on surfaces in hospitals after having previously cleaned the organic matter (35).

## Conclusion

In evaluating and comparing sporicidal activity of Bioxy products with other disinfectants against spores of *C. difficile* and *B. amyloliquefaciens*, we found that the improved method works well on all treated carriers. This is because all spores could be recovered through vortexing and membrane filtration. Surface tests demonstrate the real-life effectiveness of disinfectants in eliminating spores on surfaces. Most of the disinfectants tested meet 5 log_10_ reduction, which was our performance criterion. Further studies may be required to evaluate different concentrations of disinfectants. Another interesting approach would be combining disinfectants, thereby reducing surface destruction, increasing the safety of people using the products, and increasing the effectiveness of disinfectants.

Soil load should also be added in the spore suspensions when evaluating disinfectants for use on hospital surfaces. In addition, ASTM E2197-11 requires the use of stainless steel, but there are other types of surfaces encountered in the hospital environment, such as melamine, PMAM, glass, and arborite. This standard should therefore also be applied to assess the effectiveness of sporicides on these surfaces.

## Author Contributions

MU conceived and designed the study. RM and LY contributed to the conception of the work. MU, FG-B, and YH conceived and designed the experiments. MU performed the laboratory work, data analysis, and prepared the main manuscript. RM, YH, FG-B, and AM contributed to data analysis and revised the paper. LH supervised all the study and revised the paper.

## Conflict of Interest Statement

The authors declare that the research was conducted in the absence of any commercial or financial relationships that could be construed as a potential conflict of interest.

## References

[1] Kampf G Kramer A. Epidemiologic background of hand hygiene and evaluation of the most important agents for scrubs and rubs. Clin Microbiol Rev (2004) 17(4):863–93, table of contents. 10.1128/CMR.17.4.863-893.2004 15489352 PMC523567

[2] Rodriguez C Van Broeck J Taminiau B Delmee M Daube G. *Clostridium difficile* infection: early history, diagnosis and molecular strain typing methods. Microb Pathog (2016) 97:59–78. 10.1016/j.micpath.2016.05.018 27238460

[3] Cohen SH Gerding DN Johnson S Kelly CP Loo VG McDonald LC Clinical practice guidelines for *Clostridium difficile* infection in adults: 2010 update by the society for healthcare epidemiology of America (SHEA) and the infectious diseases society of America (IDSA). Infect Control Hosp Epidemiol (2010) 31(5):431–55. 10.1086/651706 20307191

[4] Fridkin S Baggs J Fagan R Magill S Pollack LA Malpiedi P Vital signs: improving antibiotic use among hospitalized patients. MMWR Morb Mortal Wkly Rep (2014) 63:194–200. 24598596 PMC4584728

[5] Rodriguez C Korsak N Taminiau B Avesani V Van Broeck J Delmee M *Clostridium difficile* infection in elderly nursing home residents. Anaerobe (2014) 30:184–7. 10.1016/j.anaerobe.2014.08.007 25152228

[6] Borali E Ortisi G Moretti C Stacul EF Lipreri R Gesu GP Community-acquired *Clostridium difficile* infection in children: a retrospective study. Dig Liver Dis (2015) 47(10):842–6. 10.1016/j.dld.2015.06.002 26141927

[7] Speight S Moy A Macken S Chitnis R Hoffman PN Davies A Evaluation of the sporicidal activity of different chemical disinfectants used in hospitals against *Clostridium difficile*. J Hosp Infect (2011) 79:18–22. 10.1016/j.jhin.2011.05.016 21802172

[8] Barra-Carrasco J Paredes-Sabja D. *Clostridium difficile* spores: a major threat to the hospital environment. Future Microbiol (2014) 9:475–86. 10.2217/fmb.14.2 24810347

[9] Leggett MJ McDonnell G Denyer SP Setlow P Maillard JY. Bacterial spore structures and their protective role in biocide resistance. J Appl Microbiol (2012) 113:485–98. 10.1111/j.1365-2672.2012.05336.x 22574673

[10] Kramer A Schwebke I Kampf G. How long do nosocomial pathogens persist on inanimate surfaces? A systematic review. BMC Infect Dis (2006) 6:130. 10.1186/1471-2334-6-130 16914034 PMC1564025

[11] Otter JA Yezli S Salkeld JA French GL. Evidence that contaminated surfaces contribute to the transmission of hospital pathogens and an overview of strategies to address contaminated surfaces in hospital settings. Am J Infect Control (2013) 41:S6–11. 10.1016/j.ajic.2012.12.004 23622751

[12] Comité sur les infections nosocomiales du Québec. Guide de réponse à une éclosion de diarrhée associée au Clostridium difficile en milieu hospitalier. (2014). Available from: https://www.inspq.qc.ca/pdf/publications/1964_Reponse_Eclosion_CDifficile_Hospitalier.pdf

[13] Massicotte R Désinfectants et désinfection: Principes fondamentaux. Québec: Pour le Groupe en Hygiène et salubrité du Ministère de la Santé et des Services sociaux du (2008). 75 p. Available from: http://publications.msss.gouv.qc.ca/msss/fichiers/2009/09-209-03F.pdf

[14] Massicotte R Ginestet P Yahia LH Pichette G Mafu AA Comparative study from a chemical perspective of two- and three-step disinfection techniques to control *Clostridium difficile* spores. Int J Infect Control (2011) 7:1–8. 10.3396/ijic.V7i4.031.11

[15] Leggett MJ Setlow P Sattar SA Maillard JY. Assessing the activity of microbicides against bacterial spores: knowledge and pitfalls. J Appl Microbiol (2016) 120:1174–80. 10.1111/jam.13061 26784857

[16] Mireles LK Dayan J Massicotte R Dagher F Yahia LH Interactions of active compounds of disinfectants on metallic and polymeric hospital surfaces. Clin Med Invest (2016) 1:39–47. 10.15761/CMI.1000109

[17] Dayan J Mireles LK Massicotte R Dagher F Yahia LH Effect of disinfectants on wettability and surface tension of metallic and polymeric surfaces found in hospitals. Clin Med Invest (2016) 1:48–53. 10.15761/CMI.1000110

[18] McDonnell G Russell AD. Antiseptics and disinfectants: activity, action, and resistance. Clin Microbiol Rev (1999) 12:147–79. 9880479 10.1128/cmr.12.1.147 PMC88911

[19] Perez J Springthorpe VS Sattar SA. Activity of selected oxidizing microbicides against the spores of *Clostridium difficile*: relevance to environmental control. Am J Infect Control (2005) 33:320–5. 10.1016/j.ajic.2005.04.240 16061137

[20] Wesgate R Rauwel G Criquelion J Maillard JY. Impact of standard test protocols on sporicidal efficacy. J Hosp Infect (2016) 93:256–62. 10.1016/j.jhin.2016.03.018 27133281

[21] Omidbakhsh N. Evaluation of sporicidal activities of selected environmental surface disinfectants: carrier tests with the spores of *Clostridium difficile* and its surrogates. Am J Infect Control (2010) 38:718–22. 10.1016/j.ajic.2010.02.009 21034981

[22] Fraise A. Currently available sporicides for use in healthcare, and their limitations. J Hosp Infect (2011) 77:210–2. 10.1016/j.jhin.2010.06.029 20850900

[23] Rutala WA Weber DJ The Healthcare Infection Control Practices Advisory Committee Guideline for Disinfection and Sterilization in Healthcare Facilities. (2008). 161 p. Available from: https://www.cdc.gov/infectioncontrol/pdf/guidelines/disinfection-guidelines.pdf

[24] Kuijper EJ Coignard B Tüll P Emergence of *Clostridium difficile*-associated disease in North America and Europe. Clin Microbiol Infect (2006) 12:2–18. 10.1111/j1469-0691.2006.01580.x 16965399

[25] Health Canada. Guidance Document – Safety and Efficacy Requirements for Hard Surface Disinfectant Drugs. (2014). Available from: https://www.canada.ca/content/dam/hc-sc/migration/hc-sc/dhp-mps/alt_formats/pdf/prodpharma/applic-demande/guide-ld/disinfect-desinfect/hard-surface-surfaces-dures-eng.pdf

[26] Public Health England. Identification of Bacillus Species. UK Standards for Microbiology Investigations (2015). 27 p. Available from: https://www.gov.uk/government/uploads/system/uploads/attachment_data/file/407156/ID_9i3.pdf

[27] Geeraerts S Ducatelle R Haesebrouck F Van Immerseel F. *Bacillus amyloliquefaciens* as prophylactic treatment for *Clostridium difficile*-associated disease in a mouse model. J Gastroenterol Hepatol (2015) 30:1275–80. 10.1111/jgh.12957 25800047

[28] ASTM E2197-11. Standard Quantitative Disk Carrier Test Method for Determining the Bactericidal, Virucidal, Fungicidal, Mycobactericidal and Sporicidal Activities of Liquid Chemical Germicides. West Conshohocken, PA: ASTM International (2011).

[29] Groupe de travail en Hygiène et salubrité du ministère de la Santé et des Services sociaux. Mesures d’hygiène et de salubrité au regard du Clostridium difficile, Lignes directrices. Québec: Ministère de la Santé et des Services sociaux du (2008). 22 p. Available from: http://publications.msss.gouv.qc.ca/msss/fichiers/2008/08-209-02.pdf

[30] Humphreys PN. Testing standards for sporicides. J Hosp Infect (2011) 77:193–8. 10.1016/j.jhin.2010.08.011 21122947

[31] Sorg JA Dineen SS. Laboratory maintenance of *Clostridium difficile*. Curr Protoc Microbiol (2009) 12:9A–1A. 10.1002/9780471729259.mc09a01s12 19235151

[32] ASTM E2839-11. Standard Test Method for Production of Clostridium difficile Spores for Use in Efficacy Evaluation of Antimicrobial Agents. West Conshohocken, PA: ASTM International (2014).

[33] Dagher D Ungar K Robison R Dagher F. The wide spectrum high biocidal potency of Bioxy formulation when dissolved in water at different concentrations. PLoS One (2017) 12:e0172224. 10.1371/journal.pone.0172224 28207828 PMC5313143

[34] Maillard JY. Innate resistance to sporicides and potential failure to decontaminate. J Hosp Infect (2011) 77:204–9. 10.1016/j.jhin.2010.06.028 20850897

[35] Deshaies F Ahmed D Massicotte R Pichette G Belhumeur P Mafu AA Comparison of efficacy profiles for minimum lethal concentrations (MLCs) of some commonly used commercial hospital microbicidal detergent-disinfectant products for disinfectants and sporicidal activity. Int J Infect Control (2012) 8:1–10. 10.3396/ijic.v8i2.013.12

